# Graphene Nanoplatelets/Polydimethylsiloxane Flexible Strain Sensor with Improved Sandwich Structure

**DOI:** 10.3390/s24092856

**Published:** 2024-04-30

**Authors:** Junshu Zhang, Ke Gao, Shun Weng, Hongping Zhu

**Affiliations:** 1 School of Civil and Hydraulic Engineering, Huazhong University of Science and Technology, Wuhan 430074, China; brook_zhang@hust.edu.cn (J.Z.); gaoke06@hust.edu.cn (K.G.); hpzhu@mail.hust.edu.cn (H.Z.); 2 National Center of Technology Innovation for Digital Construction, Huazhong University of Science and Technology, Wuhan 430074, China

**Keywords:** flexible sensor, graphene nanoplatelets/polydimethylsiloxane, sandwich structure, strain, size effect

## Abstract

In engineering measurements, metal foil strain gauges suffer from a limited range and low sensitivity, necessitating the development of flexible sensors to fill the gap. This paper presents a flexible, high-performance piezoresistive sensor using a composite consisting of graphene nanoplatelets (GNPs) and polydimethylsiloxane (PDMS). The proposed sensor demonstrated a significantly wider range (97%) and higher gauge factor (GF) (6.3), effectively addressing the shortcomings of traditional strain gauges. The microstructure of the GNPs/PDMS composite was observed using a scanning electron microscope, and the distribution of the conductive network was analyzed. The mechanical behavior of the sensor encapsulation was analyzed, leading to the determination of the mechanisms influencing encapsulation. Experiments based on a standard equal-strength beam were conducted to investigate the influence of the base and coating dimensions of the sensor. The results indicated that reducing the base thickness and increasing the coating length both contributed to the enhancement of the sensor’s performance. These findings provide valuable guidance for future development and design of flexible sensors.

## 1. Introduction

In civil engineering, structures are prone to cracking [1,2] and material yielding [3,4,5] over extended periods of time. The existing sensors fail to meet the corresponding measurement requirements due to their limited sensing range. In addition, sensors typically operate in diverse environments and are influenced by factors, such as temperature [6,7], humidity [8,9], and airflow [10]. Consequently, it was necessary to encapsulate and protect the sensing material. Metal foil strain gauges are the most commonly used sensors for measuring structural strain. The maximum measurable strain of metal foil strain gauges is typically 2%, which falls short of the allowable strain of steel structures of approximately 5% [11]. Consequently, if the strain of steel exceeds 2%, metal foil strain gauges cannot assess the strain state of the structures during operation. Concrete structures are prone to surface cracks, owing to their poor tensile strength [12,13,14]. If a crack occurs at the location of the strain gauge, the relatively rigid metal foil strain gauge can be easily damaged. Real-time and accurate monitoring is crucial in the field of structural health monitoring (SHM), but metal foil strain gauges are no longer sufficient to meet the requirements for large-strain measurements. This is further compounded by the fact that the base of mental foil strain gauges, typically made of PI film, tends to debond when measuring strains on uneven or complex surfaces [15,16], particularly in concrete structures where the PI film and the surface of the structure may not match well. Therefore, there is a pressing need to develop flexible sensors that offer a wide measurement range and high sensitivity for engineering applications. Flexible strain sensors appear to be a promising solution for meeting these demands [17].

Flexible piezoresistive sensors have gained extensive attention for their versatile applications. The sensors find use in various fields such as health monitoring, electronic skin, wearable electronics, and biomimetic prostheses [18,19,20,21,22,23]. One of the key advantages of flexible sensors is their high strain sensitivity coupled with excellent bendability, making them suitable for monitoring applications that involve dynamic and deformable surfaces. To ensure reliable and durable sensing performance, ductility is a crucial characteristic of strain sensors. Ductility allows the sensors to withstand repeated bending cycles without experiencing fatigue failure, thus maintaining their flexibility and consistently producing accurate sensing signals [24,25]. In addition to ductility, flexible sensors often utilize polymer blends with high dielectric constants and minimal loss for effective sensor performance [26,27,28]. Researchers have explored various sensing materials for piezoresistive applications, including graphene, carbon nanofibers, semiconductor nanowires, carbon nanotubes, and metal-/polymer-based nanocomposites [29,30,31,32,33]. For instance, the sensitivity of sensors prepared by the hierarchical synergistic structure of Au micro-cracks and carbon black nanoparticles has exceeded 2400 [34]. Flexible sensors with fast response and recovery can be prepared by stable printing methods [35]. Graphene ribbons, which are fabricated on a polymethylmethacrylate/silicon oxide substrate, have demonstrated a gauge factor of −2 at a 30% strain [36]. Similarly, graphite oxide (GO) nanoflakes, combined with polyvinylidene fluoride-conducting composites, have been developed for strain-sensing applications, achieving a gauge factor of 12.1 [37]. By attaching graphene to polymer films, the gauge factor of piezoresistive sensors can be enhanced [38]. Monolayer graphene membrane sensors have also been manufactured for electromechanical piezoresistive sensing, enabling detection limits as low as 10 Pa [39]. To further optimize the performance of touch-responsive films made of reduced graphene oxide (rGO) in IL-PDMS (ionic liquid-infused polydimethylsiloxane), researchers have incorporated imidazole IL (1-butyl-3-methylimidazolium tetrafluoroborate, BMIBF4). This combination facilitates a high rate of dispersion of rGO in IL-PDMS, thereby enhancing the usability of the rGO/IL-PDMS touch-responsive film [40]. Moreover, a composite film consisting of silver nitrate nanowires and PVA (polyvinyl alcohol) has been proposed for lateral strain-isolated ultra-sensitive pressure strain applications [37].

Graphene has great potential for SHM because of excellent electron-transfer behavior, low cost, chemical and thermal stability, sheet resistance, and low ultrahigh flexibility [41,42,43,44]. Additionally, when graphene sheets are mixed with a polymer matrix, the significant aspect ratio plays a crucial role in significantly lowering the percolation threshold [45]. Thus, efforts have been directed towards the development of transparent graphene and graphene-infused elastomers to cater to the increasing demand for flexible and stretchable electronic devices [46,47,48]. Bosque et al. [49] explained the mechanism of the effect of graphene content on the gauge factor (GF) of graphene-reinforced PDMS sensors using an RC-LRC circuit. The GF and tensile deformation of the sensor can be further improved by doping Ecoflex into graphene-enhanced polydimethylsiloxane (PDMS) [50]. Sharma et al. [51] prepared flexible sensors that can be used for human activity monitoring and health assessment using multi-modal graphene nanoparticles (GNPs)-PDMS. However, most current research on flexible electronic devices is qualitative and lacks systematic calibration, restricting their practical implementation in engineering [52,53,54]. Owing to limitations in laboratory research, many flexible sensor electrodes were affixed to both ends of the sensing specimen using a silver conductive paste. The curing of this paste resulted in increased hardness, impairing the flexibility of the sensor electrode positions. Additionally, sensors typically endure varying environmental conditions during operation, being susceptible to factors like temperature, humidity, and sunlight, necessitating encapsulation and protection. The encapsulation in a sandwich structure will influence the sensor’s force and performance. Currently, there remains insufficient research addressing the encapsulation structure of flexible sensors.

In this study, a flexible piezoresistive sensor with a high performance using GNPs and PDMS is proposed. A composite with a thickness of 1 mm and a concentration of 4.5 wt% GNPs as the dielectric layer was fabricated. Sensors with GF (>4) were tested under loading tests and could be utilized to detect large strain (>90%). To solve the problem of lead-off in the process of the test, the electrodes were formed by embedding copper foils within the composite material to extract wires. The sensor was calibrated at room temperature by using an equal-strength beam. Subsequently, the influence of the material dimensions on the encapsulation was investigated. The effects of both the base and coating on the performance of the sensor were examined using equal-strength beam experiments.

## 2. Flexible Strain Sensor with Sandwich Structure

### 2.1. Fabrication of GNPs/PDMS Nanocomposite

**Figure 1:**
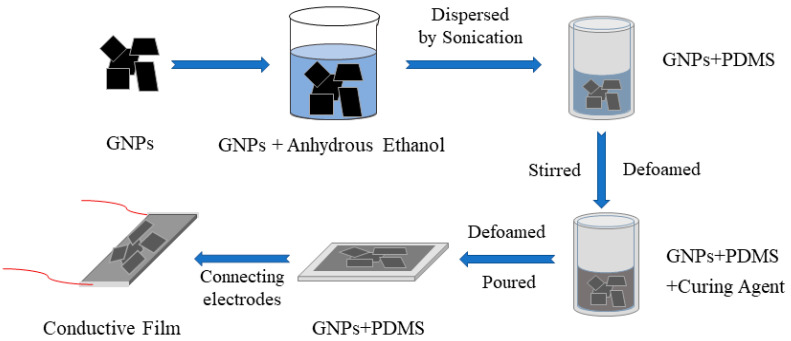
Fabrication process of the GNPs/PDMS nanocomposite.

The blending method [55,56,57,58] is commonly used to fabricate GNPs/PDMS nanocomposites. In this process, PDMS (Dow Corning Co., Ltd., Midland, TX, USA) and 99% solid content GNPs (Hanene Technology Co., Ltd., Wuhan, China) are used. Initially, anhydrous ethanol (Sinopharm Chemical Reagent Co., Ltd., Shanghai, China) is added to PDMS in a 1:1 volume ratio. The viscosity of the PDMS is reduced, and the dispersion of GNPs is enhanced through this addition. Using sonication, a highly dispersed solution is achieved by dispersing 400 mg of GNPs nanopowder in 10 mL of anhydrous ethanol. To effectively minimize the presence of macroscopic graphene clusters in the solution, the prepolymer solution is sonicated with the dispersed GNPs/anhydrous ethanol solution, facilitating the formation of the PDMS/GNPs composite in anhydrous ethanol. Before the curing process, the dispersed GNPs/base polymer emulsion is mixed with a curing agent. In order to remove air, the resulting mixture of GNPs/PDMS emulsion is placed in a vacuum chamber. After degassing, the emulsion is poured evenly into a pre-prepared Teflon mold. The solution is evaporated and heated at 50 °C for 1 h to remove the anhydrous ethanol. The composite is then solidified by baking in an oven. Typically, the PDMS specimens have an average thickness of 400 μm for the GNPs/PDMS composite. Finally, for improved electrical contact, a silver conducting epoxy is used to connect two electrodes with the composite, as depicted in Figure 1.

### 2.2. Fabrication of Encapsulated GNPs/PDMS Sensors with Sandwich Structure

**Figure 2:**
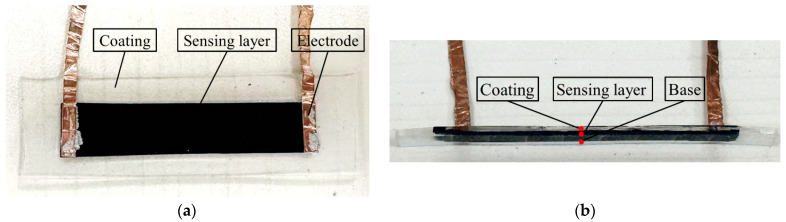
Photograph of the encapsulated GNPs/PDMS sensor: (**a**) front view; (**b**) cross-section.

The cross-sectional image of the film is displayed in Figure 2. A partially cured PDMS substrate was initially prepared on a Teflon substrate and allowed to cure at room temperature for 48 h. Subsequently, the pre-cured GNPs/PDMS film was coated with a PDMS slurry mixture and further incubated at room temperature for an additional 48 h. The purpose is to minimize the influence of different temperature characteristics between the GNPs/PDMS composite and the PDMS. The middle conductive film had a thickness of 200 μm, whereas the sandwich structure layer had a thickness of 300 μm. Finally, the encapsulated GNPs/PDMS sensor was obtained by separating the sandwich structure from the Teflon substrate.

### 2.3. Morphological, Electrical, and Piezoresistive Characterization

The surface and cross-sectional morphologies of the GNPs/PDMS composite were determined using scanning electron microscopy (SEM, Sirion 200, FEI Co., Ltd., Eindhoven, The Netherlands). A two-point probe Keithey 7510 multimeter (Sinopharm Tektronix Co., Ltd., Beaverton, OR, USA) was used to determine the dielectric properties of the GNPs/PDMS sensor within a frequency of 30 Hz at ambient temperature. The data collection involved the use of parallel plate probes. The samples were placed between parallel plate electrodes, and the data were recorded during this process. The sensor sensitivity was evaluated using GF, provided by the formula GF = (Δ*R*/*R*_0_)/*ε*, where Δ*R*/*R*_0_ is the electrical resistance change ratio, Δ*R* = *R* − *R*_0_ is the real-time change in the electrical resistance, *R*_0_ is the initial resistance, *R* is the testing resistance, and *ε* is the strain.

In preparation for performance testing under uniaxial cyclic tensile conditions, the encapsulated GNPs/PDMS sensor was fastened to the displacement table (Electronic universal material testing machine C45.105EYMTS, Systems Co., Ltd., Shanghai, China) using a fixture. To ensure that the transducer was predominantly in tension during cycling, the fixture was adjusted to create a 5000 με prestrain on the sensor. Next, the electrodes were attached to a copper wire, and a Keithey 7510 multimeter was connected to the sensor via a copper wire for data acquisition. The range and display accuracy of the multimeter were adjusted according to the initial resistance of the sensor to ensure accurate data display. Cyclic tensile tests were performed on the sensor, with successive application of strains of 5%, 10%, 15%, and 20%.

## 3. Characteristics

### 3.1. Morphology

The penetration threshold of GNP was experimentally obtained to be 3 wt%, at which point the conductive pathway within the composite was incomplete. As the mass fraction of GNPs increased, the internal conductive network within the sensors became denser, leading to a decrease in the resistance. However, owing to the hindrance caused by GNPs, the cross-linking degree of PDMS decreases and the porosity of the GNPs/PDMS composite increases, resulting in uncontrollable cracks on the material surface and influencing material properties. Therefore, the microscopic structures of the GNPs/PDMS films with mass fractions of 4 wt% and 5 wt% GNPs were chosen for comparison.

**Figure 3:**
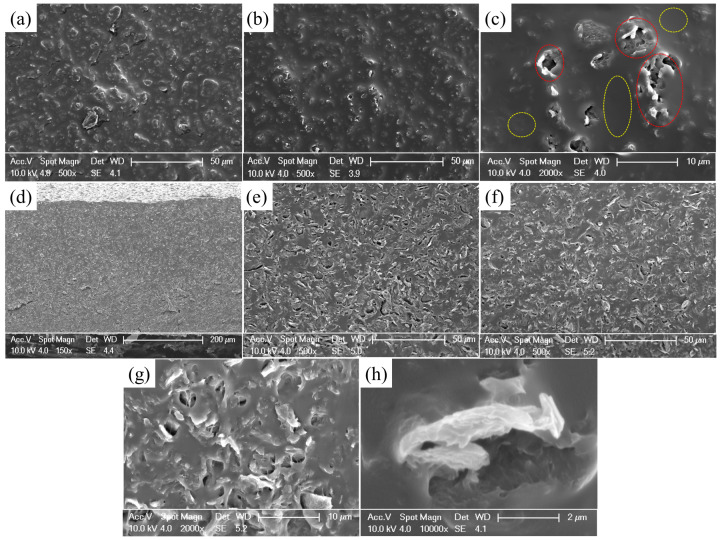
SEM image (**a**) Surface of 5 wt% GNPs (**b**) Surface of 4 wt% GNPs (**c**) Enlarged inset of the surface (**d**) Macrographic appearance of the cross-section (**e**) Cross-section of 5 wt% GNPs (**f**) Cross-section of 4 wt% GNPs (**g**) Enlarged inset of the cross-section (**h**) GNPs embedded in PDMS.

Figure 3a,b,e,f show the SEM images of the GNPs blended with ratios of 5 wt% and 4 wt%, respectively. SEM was used to characterize the surface morphologies of the GNPs/PDMS films, as shown in Figure 3a–c. The GNPs were completely covered with PDMS. Owing to the accumulation of GNPs, PDMS did not fill the pores, forming a micropore structure. The yellow circle in Figure 3c represents the dense PDMS after vacuuming, whereas the red circle represents the micropores. The images show that the GNPs are uniformly distributed at the interface of the PDMS regions and tend to aggregate in certain areas, forming an interconnected 3D nanostructured network. Additionally, cross-sectional morphologies of the GNPs in the GNPs/PDMS composite were observed. As shown in Figure 3d–f, throughout the GNPs/PDMS composite, a skeleton composed of GNPs was present, with the GNPs positioned at the interfaces of the PDMS phase, creating a densely interconnected structure. The interstitial spaces between PDMS are occupied by GNPs due to the excluded volume effects of latex particles. From Figure 3, it can be observed that the pores are distributed around the GNPs, and the PDMS that is not in contact with the GNPs has almost no pores.

### 3.2. Electrical and Mechanical Properties

**Figure 4:**
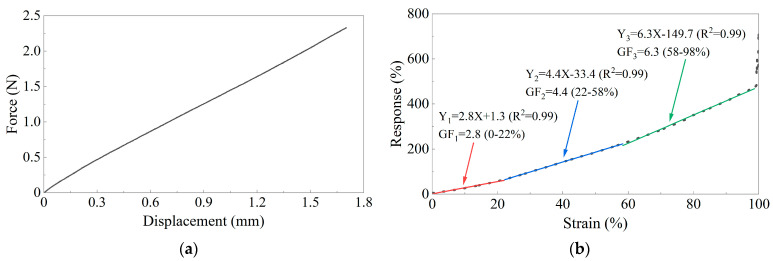
Uniaxial tensile test results: (**a**) Force-displacement curve of the GNPs/PDMS sample; (**b**) Resistance-strain curve of the GNPs/PDMS sample.

Different mass fractions were used to investigate the conductivity of the GNPs/PDMS composite, as illustrated in Figure 4a. As anticipated, the sample with a GNP mass fraction of 3 wt% displayed exceptionally low conductivity, resembling that of an insulator. In contrast, the remaining samples exhibited favorable electrical characteristics. The magnitude of electrical conductivity is greatly influenced by the mass fraction. With the mass fraction of GNPs increasing, the conductive network gradually became denser and more distinct, leading to a significant enhancement in electrical conductivity from 7 × 10^−9^ S/cm (3 wt%) to 0.0026 S/cm (6 wt%). However, when the mass fraction exceeded 4 wt%, the conductivity did not show a significant increase. On the surface of the PDMS matrix, a stable and saturated conductive network is observed.

First, the mechanical properties of the flexible strain sensors were investigated through an axial loading experiment. The film thicknesses were measured at different locations. The specimen was fixed with a universal testing machine, and the specimen was straight and stress-free. The machine started to load at a fixed velocity until the specimen broke, and the resistance of the specimen was continuously measured during the entire process.

The average film thickness of the GNPs/PDMS samples was 0.57 mm. In engineering applications, structural strains typically do not exceed 5%. The fixture displacement and tension data of five groups of GNPs/PDMS samples from tensile to 10% strain by the universal testing machine were measured. The average of the five groups of calculated strain data was used to draw the stress–strain curve of the GNPs/PDMS film (Figure 4a). Within the range of the 10% strain, the force-displacement curve of the specimen exhibited well-defined linearity. The Young’s modulus was approximately 5.84 MPa. The GNPs/PDMS sample possessed good ductility and stability to meet the requirements for large-strain sensing.

Figure 4b shows that during monotonic stretching tests, the resistance of the encapsulated GNPs/PDMS sensor exhibits a nearly linear variation with strain, but there is a slight curvature within a small strain range. The elongation at fracture was 34.116 mm, and the initial gauge length of the sensor was 35 mm. Therefore, the mechanical tensile strain was calculated to be 97.47%. The variation of sensor resistance with strain was triple linear and the R^2^ of the fitting line were all greater than 0.99, with GFs of 2.8, 4.4, and 6.3, respectively, which were higher than that of the metal foil strain gauges.

**Figure 5:**
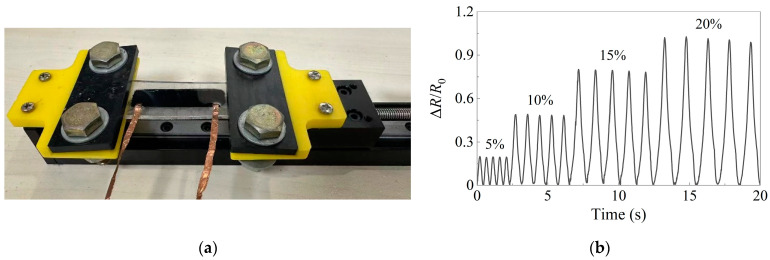
Dynamic stretch–release cycle response of the encapsulated GNPs/PDMS sensor for various strains from 5% to 20%: (**a**) Uniaxial cyclic tensile test of the sensor; (**b**) Resistance variation of the sensor in cyclic tensile test.

The resistance of the encapsulated sensor was evaluated under various strains. Figure 5 demonstrates the variations in the sensor’s resistance measurements under cyclic tensile strains of 5%, 10%, 15%, and 20%. The observed resistance variations at different strains were significantly distinct, indicating the enhanced accuracy of the strain sensor in detecting corresponding strains. The resistance changes remained consistent during five repetitive cycles at the same strain level, indicating the stability of the sensor. Additionally, the relative changes in resistance of the sensor remained stable, further demonstrating its reliable performance. Importantly, each peak displayed a similar shape and height, serving as an indication of durability. In addition, the sensor has properties such as response/recovery time and repeatability. In this paper, the main object is the relationship between resistance change and strain, and other properties will not be discussed for the time being.

## 4. Size Effects

To explore the size effect, a laboratory test was conducted in which the flexible strain sensor was attached to an equal-strength beam. The influence of base thickness and coating length on the relative resistance was examined through experiments. [59].

**Figure 6:**
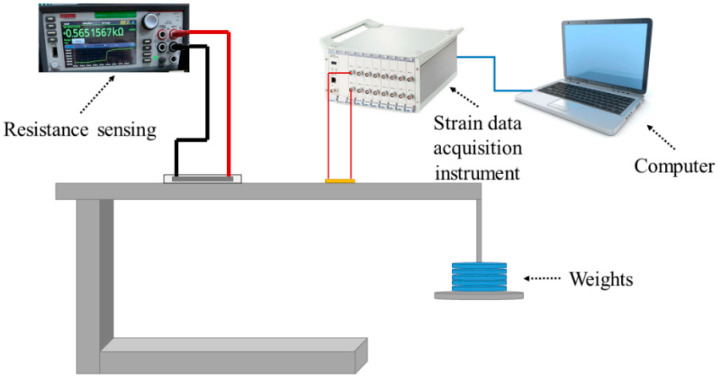
Strain test system.

Figure 6 shows the test setups of equal-strength beam tests. A uniform strain is achieved along the beam with a force. The tests minimize measurement discrepancies arising from different strains along the beam, thus enabling a more accurate assessment of the size effect on the performance of flexible sensors. To increase strain on the beam, weights were added to the tray at the end of the beam during the loading test. The strain was evaluated by GNPs/PDMS strain sensors and metal foil strain gauges.

### 4.1. Base Thickness

The GNPs/PDMS strain sensors with different base thicknesses were fabricated using the same process. After equal-strength beam tests, the sensors were removed using a solvent, and the base thicknesses were measured to be 0.28 mm, 1.22 mm, and 2.19 mm, respectively.

**Figure 7:**
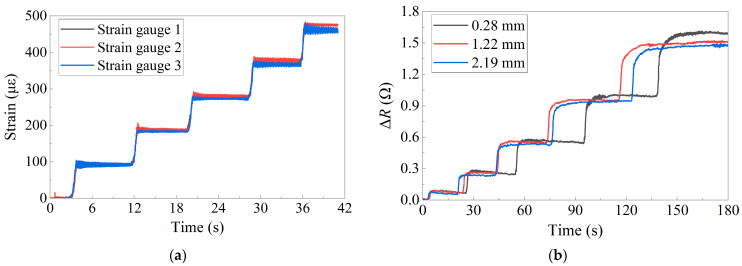
Data from loading test: (**a**) Metal foil strain gauges; (**b**) Flexible strain sensors.

Figure 7 depicts the results of the loading test conducted on flexible strain sensors and metal foil strain gauges with varying base thicknesses. Initially, an approximate increase of 90 με in strain was produced on the beam with equal strength at each loading step. Figure 7a demonstrates the consistent linear growth in readings obtained from the calibrated metal foil strain gauges. Conversely, the data acquired from flexible sensors demonstrated a nonlinear growth. When comparing the two types of sensors, the flexible sensor displayed a progressive increase with the growth of deformation, eventually reaching stability, whereas the metal foil strain gauges maintained a consistent linear growth pattern. The hysteresis effect can be ascribed to the flexible material properties of the sensor, resulting in the slow variation. With an increase in the applied load, the impact of hysteresis became more pronounced. Overall, the measurement data obtained from two kinds of sensors exhibited stability. Furthermore, the initial resistance of the sensor is 399.25 Ω. As the thickness increases, there is a slight change in the initial resistance value with a magnitude of about 5 Ω. For comparison, the curve of the relative change in the resistance with strain is taken. Figure 7b indicates a gradual decrease in resistance variation with an increase in base thickness.

**Figure 8:**
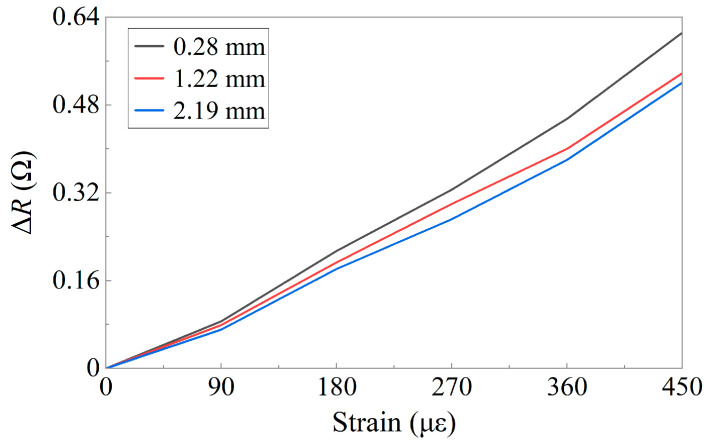
Resistance variation for sensors with different base thickness.

Figure 8 illustrates the resistance variation of the flexible strain sensors with different base thicknesses after stabilization at various strains. The results indicate that the sensors of three different sizes exhibit the same trend in resistance variation with respect to strain and are capable of detecting small deformations in structures. However, with an increase in the base thickness, the resistance variation of the different sensors under the same strain varies. The initial resistance *R*_0_ of the flexible sensor was fixed, and according to the formula for GF, it was determined by Δ*R*. For the same strain, a larger Δ*R* led to a higher GF for the sensor. Taking the measurement results of the sensor with a thickness of 2.19 mm as a reference, the GF of the sensor with a 1.22 mm base thickness increased by 4%, and when the base thickness decreased to 0.28 mm, the GF increased by 10%. Hence, it is advisable to reduce the base thickness during sensor encapsulation.

### 4.2. Coating Length

**Figure 9:**
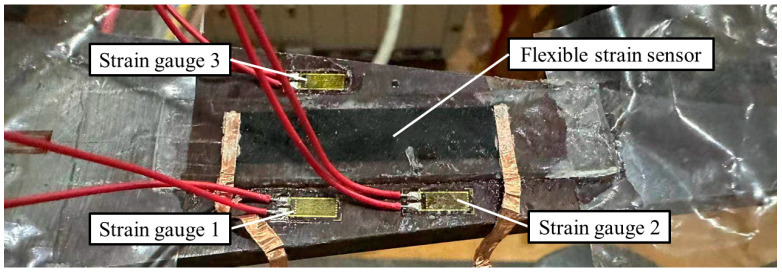
Sensors bonded with different coating length.

The flexible sensor was divided into three segments and sequentially adhered to an equal-strength beam with an epoxy resin. First, the sensing layer segment of the sensor was adhered to the upper surface of the beam with a plastic film placed underneath at both ends of the coating. Only the sensing layer segment is affixed to the beam because the epoxy resin cannot bond with the plastic film. The coatings at both ends are not affected by the beam and can be considered as the sensing layer unaffected by the coating. Simultaneously, metal foil strain gauges were adhered to the flexible sensor for comparison. After the loading test, half of the coatings at both ends of the flexible sensor were adhered to the beam using the same method. In this case, because of the traction transferred by the beam to the coatings at the ends, tension was applied to the sensing layer, thereby altering the rate of resistance change in the flexible sensor. Finally, the flexible sensor fully adhered to the equal-strength beam, and the coating length was further increased. This allowed for a comparison of the resistance changes induced by the loading test. The bonding method of the sensors is illustrated in Figure 9.

**Figure 10:**
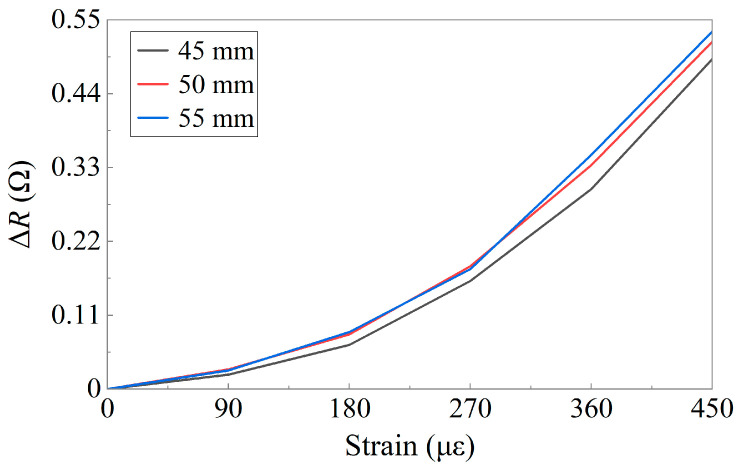
Resistance variation for sensors with different coating length.

The relationship between the relative resistance change and the strain is shown in Figure 10. The resistance variation followed a pattern similar to that of a previous experiment. Altering the length of the coating also affected the sensitivity of the sensor. Taking the measurement results of the sensor with a length of 45 mm as a reference, the GF of the sensor with a 50 mm coating length increased by 10%, and when the coating length increased to 55 mm, the GF increased by 12%. When the coating length matched that of the sensing layer, with no coating protection at the ends of the sensing layer, the variation in Δ*R* was minimal. As the coating length increased, the GF of the sensor improved. This phenomenon primarily arises from changes in the stress state of the sensing layer owing to variations in the coating length. In cases where there is no coating protection at the ends, the sensing layer experiences free-boundary conditions. When coatings are present at both ends, they also undergo deformation from the measured structure, exerting a force on the ends of the sensing layer, resulting in more pronounced deformation and an increase in Δ*R*. Therefore, appropriately increasing the coating length helps fully utilize the performance of the sensor.

Additionally, experiments were conducted by varying the coating thickness. However, the sensor performance remained largely unchanged. Further elaboration of this point has been omitted. These observations underscore the influence of the encapsulation structure on the sensor performance, necessitating optimization.

## 5. Conclusions

In this study, an encapsulated GNPs/PDMS sensor with stable conductive and sensing network features was developed with simplicity and low cost. The mechanical and sensing characteristics were investigated from microscopic to macroscopic aspects. In the working range of the sensor (0% < ε < 97%), the GF can reach 4.87, which is significantly higher than that of the metal foil strain gauge. The elastic modulus of the GNPs/PDMS composite material was approximately 5.84 MPa, indicating its excellent flexibility. The resistance of the sensor was characterized by observing its microstructure using SEM.

A study was conducted to examine the influence of the dimensions of the encapsulation material on the performance of the sensor. The experimental results demonstrated that appropriately adjusting the dimensions of the encapsulation material contributed to optimizing the sensor performance. When encapsulating and protecting the sensing layer, reducing the thickness of the base is advisable; otherwise, it may diminish sensor GF. Furthermore, increasing the coating length of the sensor can enhance GF. These results provide a valuable reference for the design and development of flexible strain sensors for deformation monitoring.

## Data Availability

The data used to support the findings of this study are available from the corresponding author upon request.

## References

[1] Zhou Y., Lian H., Li Z., Yin L., Ji Q., Li K., Qi F., Huang Y. (2022). Crack Engineering Boosts the Performance of Flexible Sensors. VIEW.

[2] Zheng J., Liu Y., Luo R., Liu H., Zhou Z., He J. (2024). A Subpixel Concrete Crack Measurement Method Based on the Partial Area Effect. Buildings.

[3] Hu R., Hu S., Yang M., Zhang Y. (2022). Metallic Yielding Dampers and Fluid Viscous Dampers for Vibration Control in Civil Engineering: A Review. Int. J. Struct. Stab. Dyn..

[4] Xu Y., Cui T., Wu B., Wang Z., Song Y. (2023). Dynamic Mode I Fracture Characteristics of Jute Fiber-Reinforced Rubber Mortar. Eng. Fract. Mech..

[5] Guo B., Lin X., Wu Y., Zhang L. (2023). Performance of Compression Yielded FRP-Reinforced Concrete Beams with T Sections. J. Compos. Constr..

[6] Liu Z., Tian B., Jiang Z., Li S., Lei J., Zhang Z., Liu J., Shi P., Lin Q. (2023). Flexible Temperature Sensor with High Sensitivity Ranging from Liquid Nitrogen Temperature to 1200 °C. Int. J. Extrem. Manuf..

[7] Wang B., Cai H., Jia Q., Pan H., Li B., Fu L. (2023). Smart Temperature Sensor Design and High-Density Water Temperature Monitoring in Estuarine and Coastal Areas. Sensors.

[8] Lin X., Xue H., Li F., Zhao H., Zhang T. (2024). An Electrolyte-Mediated Paper-Based Humidity Sensor Fabricated by an Office Inkjet Printer. IEEE Electron Device Lett..

[9] Zhang M., Duan Z., Zhang B., Yuan Z., Zhao Q., Jiang Y., Tai H. (2023). Electrochemical Humidity Sensor Enabled Self-Powered Wireless Humidity Detection System. Nano Energy.

[10] Liu Y., Liu Z., Gao K., Huang Y., Zhu C. (2022). Efficient Graphical Algorithm of Sensor Distribution and Air Volume Reconstruction for a Smart Mine Ventilation Network. Sensors.

[11] Babkin S.E., Il’yasov R.S. (2010). On the Possibility of Estimating the Elasticity Limit and Residual Deformations in Ferromagnetic Metals Using the Parameters of Electromagnetic-Acoustic Transformation. Russ. J. Nondestruct. Test..

[12] Yang X., Wang Z., Su P., Xie Y., Yuan J., Zhu Z. (2021). A Method for Detecting Metal Surface Cracks Based on Coaxial Resonator. IEEE Sens. J..

[13] Wang X., Su P., Zou J., Wu J., Yang X. (2022). Detection of Metallic Surface Cracks Based on Multiunit Periodic Resonant Structure. IEEE Sens. J..

[14] Pang Q., Dong G., Yang X. (2023). Metal Crack Detection Sensor Based on Microstrip Antenna. IEEE Sens. J..

[15] Kim H. (2006). Closed Form Solution for Strain Energy Release Rate Distribution in Debonded One-Edge Free Postbuckled Composite Flanged Joints. Compos. Sci. Technol..

[16] Lau K., Chan C., Zhou L., Jin W. (2001). Strain Monitoring in Composite-Strengthened Concrete Structures Using Optical Fibre Sensors. Compos. Part B Eng..

[17] Gao K., Zhang Z., Weng S., Zhu H., Yu H., Peng T. (2022). Review of Flexible Piezoresistive Strain Sensors in Civil Structural Health Monitoring. Appl. Sci..

[18] Gao Y., Yu L., Yeo J.C., Lim C.T. (2020). Flexible Hybrid Sensors for Health Monitoring: Materials and Mechanisms to Render Wearability. Adv. Mater..

[19] Chen J., Zhu Y., Chang X., Pan D., Song G., Guo Z., Naik N. (2021). Recent Progress in Essential Functions of Soft Electronic Skin. Adv. Funct. Mater..

[20] Alshawabkeh M., Alagi H., Navarro S.E., Duriez C., Hein B., Zangl H., Faller L.-M. (2023). Highly Stretchable Additively Manufactured Capacitive Proximity and Tactile Sensors for Soft Robotic Systems. IEEE Trans. Instrum. Meas..

[21] Liu Y., Wang H., Zhao W., Zhang M., Qin H., Xie Y. (2018). Flexible, Stretchable Sensors for Wearable Health Monitoring: Sensing Mechanisms, Materials, Fabrication Strategies and Features. Sensors.

[22] Kim H., Kwon Y., Lim H., Kim J., Kim Y., Yeo W. (2021). Recent Advances in Wearable Sensors and Integrated Functional Devices for Virtual and Augmented Reality Applications. Adv. Funct. Mater..

[23] Bora M., Kottapalli A.G.P., Miao J., Triantafyllou M.S. (2017). Biomimetic Hydrogel-CNT Network Induced Enhancement of Fluid-Structure Interactions for Ultrasensitive Nanosensors. NPG Asia Mater..

[24] Wang P., Takagi T., Takeno T., Miki H. (2013). Early Fatigue Damage Detecting Sensors—A Review and Prospects. Sens. Actuators A Phys..

[25] Persons A.K., Ball J.E., Freeman C., Macias D.M., Simpson C.L., Smith B.K., Burch V.R.F. (2021). Fatigue Testing of Wearable Sensing Technologies: Issues and Opportunities. Materials.

[26] Zhu Y., Huang X., Tian Y., Ji C., Cao W., Zhao L. (2018). Experimental Study on the Icing Dielectric Constant for the Capacitive Icing Sensor. Sensors.

[27] Hao H., Wang D., Wang Z., Yin B., Ruan W. (2020). Design of a High Sensitivity Microwave Sensor for Liquid Dielectric Constant Measurement. Sensors.

[28] Zhong W., Wang D., Ke Y., Ming X., Jiang H., Li J., Li M., Chen Q., Wang D. (2024). Multi-Layer Polyurethane-Fiber-Prepared Entangled Strain Sensor with Tunable Sensitivity and Working Range for Human Motion Detection. Polymers.

[29] Fu M., Ye Y., Niu Y., Guo S., Wang Z., Liu X. (2024). Graphene-Based Tunable Dual-Frequency Terahertz Sensor. Nanomaterials.

[30] Mostafa M.H., Ali E.S., Darwish M.S.A. (2022). Polyaniline/Carbon Nanotube Composites in Sensor Applications. Mater. Chem. Phys..

[31] Wang H., Cao H., Wu H., Zhang Q., Mao X., Wei L., Zhou F., Sun R., Liu C. (2023). Environmentally Friendly and Sensitive Strain Sensor Based on Multiwalled Carbon Nanotubes/Lignin-Based Carbon Nanofibers. ACS Appl. Nano Mater..

[32] Li Z., Huang H., Zhao D., Chen S. (2023). A Reliable Strain Sensor Based on Bridging GaN Nanowires. IEEE Sens. J..

[33] He K., Xing S., Shen Y., Jin C. (2022). A Flexible Optical Gas Pressure Sensor as the Signal Readout for Point-of-Care Immunoassay. Analyst.

[34] Zhou R., Zhang Y., Xu F., Song Z., Huang J., Li Z., Gao C., He J., Gao W., Pan C. (2023). Hierarchical Synergistic Structure for High Resolution Strain Sensor with Wide Working Range. Small.

[35] Huang Q., Jiang Y., Duan Z., Yuan Z., Wu Y., Peng J., Xu Y., Li H., He H., Tai H. (2023). A Finger Motion Monitoring Glove for Hand Rehabilitation Training and Assessment Based on Gesture Recognition. IEEE Sens. J..

[36] Wang Y., Yang R., Shi Z., Zhang L., Shi D., Wang E., Zhang G. (2011). Super-Elastic Graphene Ripples for Flexible Strain Sensors. ACS Nano.

[37] Eswaraiah V., Balasubramaniam K., Ramaprabhu S. (2012). One-Pot Synthesis of Conducting Graphene–Polymer Composites and Their Strain Sensing Application. Nanoscale.

[38] Li R., Zhang Q., Zhao E., Li J., Gu Q., Gao P. (2019). Etching- and Intermediate-Free Graphene Dry Transfer onto Polymeric Thin Films with High Piezoresistive Gauge Factors. J. Mater. Chem. C.

[39] Wang Z., Guo S., Li H., Wang B., Sun Y., Xu Z., Chen X., Wu K., Zhang X., Xing F. (2019). The Semiconductor/Conductor Interface Piezoresistive Effect in an Organic Transistor for Highly Sensitive Pressure Sensors. Adv. Mater..

[40] Ponnamma D., Sadasivuni K.K., Cabibihan J.-J., Yoon W.J., Kumar B. (2016). Reduced Graphene Oxide Filled Poly(Dimethyl Siloxane) Based Transparent Stretchable, and Touch-Responsive Sensors. Appl. Phys. Lett..

[41] Duan L., D’hooge D.R., Cardon L. (2020). Recent Progress on Flexible and Stretchable Piezoresistive Strain Sensors: From Design to Application. Prog. Mater. Sci..

[42] Wang Y., Hu S., Xiong T., Huang Y., Qiu L. (2022). Recent Progress in Aircraft Smart Skin for Structural Health Monitoring. Struct. Health Monit..

[43] Yang H., Xue T., Li F., Liu W., Song Y. (2019). Graphene: Diversified Flexible 2D Material for Wearable Vital Signs Monitoring. Adv. Mater. Technol..

[44] Qureshi A., Niazi J.H. (2023). Graphene-Interfaced Flexible and Stretchable Micro–Nano Electrodes: From Fabrication to Sweat Glucose Detection. Mater. Horiz..

[45] McAllister M.J., Li J.-L., Adamson D.H., Schniepp H.C., Abdala A.A., Liu J., Herrera-Alonso M., Milius D.L., Car R., Prud’homme R.K. (2007). Single Sheet Functionalized Graphene by Oxidation and Thermal Expansion of Graphite. Chem. Mater..

[46] Del Bosque A., Sánchez-Romate X., Sánchez M., Ureña A. (2022). Wearable Sensors Based on Graphene Nanoplatelets Reinforced Polydimethylsiloxane for Human Motion Monitoring: Analysis of Crack Propagation and Cycling Load Monitoring. Chemosensors.

[47] He S., Zhang Y., Gao J., Nag A., Rahaman A. (2022). Integration of Different Graphene Nanostructures with PDMS to Form Wearable Sensors. Nanomaterials.

[48] Liu A., Ni Z., Chen J., Huang Y. (2020). Highly Sensitive Graphene/Polydimethylsiloxane Composite Films near the Threshold Concentration with Biaxial Stretching. Polymers.

[49] Bosque A.D., Sánchez-Romate X.F., Sánchez M., Ureña A. (2022). Ultrasensitive and Highly Stretchable Sensors for Human Motion Monitoring Made of Graphene Reinforced Polydimethylsiloxane: Electromechanical and Complex Impedance Sensing Performance. Carbon.

[50] Del Bosque A., Sánchez-Romate X.F., Gómez A., Sánchez M., Ureña A. (2023). Highly Stretchable Strain Sensors Based on Graphene Nanoplatelet-Doped Ecoflex for Biomedical Purposes. Sens. Actuators A Phys..

[51] Sharma P., Sharma R., Janyani V., Verma D. (2023). Development of a Multi-Modal Graphene Nanoparticles (GNP)- Polydimethylsiloxane (PDMS) Flexible Sensor for Human Activity Monitoring and Health Assessment. Int. J. Electrochem. Sci..

[52] Li J., Wang P., Han X., Zhao T., Yoon S. (2023). Strategies for Sensor Virtual In-Situ Calibration in Building Energy System: Sensor Evaluation and Data-Driven Based Methods. Energy Build..

[53] Ma H., Yao S., Xing Y. (2022). Redundant Parallel Beam Multiaxis Force Sensor—Accuracy Space. IEEE Sens. J..

[54] Fouad K.M., Hassan B.M., Salim O.M. (2022). Hybrid Sensor Selection Technique for Lifetime Extension of Wireless Sensor Networks. Comput. Mater. Contin..

[55] Yi Y., Chiao M., Mahmoud K.A., Wu L., Wang B. (2022). Preparation and Characterization of PVA/PVP Conductive Hydrogels Formed by Freeze–Thaw Processes as a Promising Material for Sensor Applications. J. Mater. Sci..

[56] Heredia-Rivera U., Gopalakrishnan S., Kadian S., Nejati S., Kasi V., Rahimi R. (2022). A Wireless Chipless Printed Sensor Tag for Real-Time Radiation Sterilization Monitoring. J. Mater. Chem. C.

[57] Pervin S., Sathiyanathan P., Prabu A.A., Kim K.J. (2022). Piezoelectric Sensor Based on Electrospun Poly(Vinylidene Fluoride)/Sulfonated Poly(1,4-phenylene Sulfide) Blend Nonwoven Fiber Mat. J. Appl. Polym. Sci..

[58] Asghari N., Hassanian-Moghaddam D., Javadi A., Ahmadi M. (2023). Enhanced Sensing Performance of EVA/LDPE/MWCNT Piezoresistive Foam Sensor for Long-Term Pressure Monitoring. Chem. Eng. J..

[59] Weng S., Zhang J., Yan Z., Gao K., Chen Z., Wu L. (2024). Improved Strain Transfer Model for Flexible Sensors Based on Non-Uniform Distribution of Shear Stress in Each Layer. Measurement.

